# Effects of Ploidy and Recombination on Evolution of Robustness in a Model of the Segment Polarity Network

**DOI:** 10.1371/journal.pcbi.1000296

**Published:** 2009-02-27

**Authors:** Kerry J. Kim, Vilaiwan M. Fernandes

**Affiliations:** Center for Cell Dynamics, Friday Harbor Labs, University of Washington, Friday Harbor, Washington, United States of America; University of Texas at Austin, United States of America

## Abstract

Many genetic networks are astonishingly robust to quantitative variation, allowing these networks to continue functioning in the face of mutation and environmental perturbation. However, the evolution of such robustness remains poorly understood for real genetic networks. Here we explore whether and how ploidy and recombination affect the evolution of robustness in a detailed computational model of the segment polarity network. We introduce a novel computational method that predicts the quantitative values of biochemical parameters from bit sequences representing genotype, allowing our model to bridge genotype to phenotype. Using this, we simulate 2,000 generations of evolution in a population of individuals under stabilizing and truncation selection, selecting for individuals that could sharpen the initial pattern of engrailed and wingless expression. Robustness was measured by simulating a mutation in the network and measuring the effect on the engrailed and wingless patterns; higher robustness corresponded to insensitivity of this pattern to perturbation. We compared robustness in diploid and haploid populations, with either asexual or sexual reproduction. In all cases, robustness increased, and the greatest increase was in diploid sexual populations; diploidy and sex synergized to evolve greater robustness than either acting alone. Diploidy conferred increased robustness by allowing most deleterious mutations to be rescued by a working allele. Sex (recombination) conferred a robustness advantage through “survival of the compatible”: those alleles that can work with a wide variety of genetically diverse partners persist, and this selects for robust alleles.

## Author Summary

Most so-called “higher organisms” are diploid (have two copies of each gene) and reproduce sexually. Diploidy may be advantageous if one functional copy can mask the effects of a mutation in the other copy; however, it is a liability if most mutations are dominant. Sex can increase genetic diversity and the rate of evolution by creating new combinations of alleles that might function better together but can also disrupt working combinations. Given these trade-offs, why are sex and diploidy so common, and why do they occur so often together? We hypothesize that sex and diploidy allow gene networks to evolve to function more robustly in the face of genetic and environmental variation. This robustness would be advantageous because organisms are exposed to constantly changing environments and all genes undergo mutation. To test this hypothesis, we simulated evolution in a model of the segment polarity network, a well-studied group of genes essential for proper development in many organisms. We compared the robustness of haploid and diploid populations that reproduced either sexually or asexually. Sexually reproducing diploid populations evolved the greatest robustness, suggesting an explanation for the selective advantage of diploid sexual reproduction.

## Introduction

Phenotypic robustness, also called canalization [1], is the ability of a phenotype to persist when challenged by a perturbation to the system producing it. Many phenotypes are not the product of an individual gene, but rather arise from interactions within larger gene networks. The functions of several well-studied networks have been shown or predicted to be robust to quantitative variation in the biochemical kinetics [2]–[8]. This variation can come from both intrinsic (genetic) and extrinsic (environmental) sources: Genetic diversity (polymorphism) within populations can produce variation in gene expression levels and in the activity of gene products [9]–[13]. In a genetically diverse, sexually reproducing population, recombination is continuously producing new combinations of alleles, and robustness to genetic variation would confer a fitness advantage. This intuition is supported by experiments showing much genetic variation is hidden—i.e. quantitative variation between individuals has no detectable effect on phenotype [10]. Another source of perturbation is environmental: Individuals can transiently experience a broad range of potentially noxious environments (due to pH, oxygen level, starvation conditions, or temperature) that alter protein activity and potentially disrupt gene networks. While only genetic effects are heritable, genetic and environmental variation both perturb network dynamics, and robustness to one may confer robustness to the other [14]–[16].

A possible mechanism to increase robustness is diploidy, as mutations can be masked by a functional copy (a recessive mutation), allowing greater tolerance to mutation. However, it is unclear whether diploidy is an advantage in genetic networks, because it is also potentially harmful: a diploid network will have mutations twice as often as a haploid, and a single bad allele could break the network (a dominant mutation). Most deleterious mutations in enzyme coding genes are recessive to the wild type alleles [17]–[19]. For metabolic networks, Kacser and Burns [20] showed theoretically that most mutations are recessive because in long metabolic pathways each individual enzyme contributes weakly to the total flux. This theory was formulated for metabolic networks where all gene products were enzymes, and it may not hold for gene regulatory networks [21],[22]. Importantly, a majority of disease-causing mutations in transcription factors are dominant [23]. Experimental evolution on yeast, which can exist either as haploids or diploids, has shown that different ploidies are advantageous under different conditions [24]–[26]. The advantage of diploidy depends on the frequency of deleterious dominant mutations, mutation rates, and other factors [27]–[30]. However, this is an oversimplification because if most deleterious mutations are recessive, the evolutionary advantage of diploidy remains questionable as the effects of rare beneficial recessive mutations could likewise be masked. Such masking of beneficial mutations in a diploid population has been observed in antibiotic resistance evolution in yeast [25]. Thus, models investigating the effects of ploidy on robustness need to incorporate both the spectrum of possible mutations, and the functional context in which they occur (e.g. participation in a network).

Theory predicts that genetic variation combined with gene interaction favors the evolution of phenotypic robustness [14],[31],[32]. The evolution of increased robustness to mutation (mutational robustness) has been predicted by models of RNA folding [33],[34] and randomly wired transcriptional networks [5],[35],[36]. Theory and modeling predict that sexually reproducing populations, with recombination shuffling alleles, should experience stronger selection for robustness than asexual populations [37], and has been shown to hold for randomly-wired interaction networks [35]. However, it is unknown whether these results hold for real networks because interactions between mutations may be more complicated than theoretical studies assume. Additionally, real networks may have subtle topological or regulatory architecture that differ from randomly-wired model networks in important ways. Sex and diploidy are commonly found together, and both may produce greater robustness, but this has not been tested for gene regulatory networks.

In this study, we investigate how ploidy and sex (recombination) affect the evolution of robustness in a detailed model of the segment polarity network. Previous modeling studies focused on highly simplified and abstract networks [5], [33]–[36], and it is essential to test whether these findings hold in a realistic network with a known function. The segment polarity network is a canonical example of a pattern forming network that is robust to variation in its underlying biochemical kinetics [2],[38]. It is essential for development in many insects, and the function of its genes and their interactions within the network are well-understood. In this network, gene expression is regulated at both pre- and post-transcriptional levels, with some regulations requiring cell–cell communication. During development prior to the operation of the segment polarity network, gap and pair-rule genes activate expression of wg and en in a noisy prepattern of stripes. The segment polarity network in *Drosophila* development then sharpens and maintains these stripes through the lifetime of the organism. Correct location of these stripes of expression is essential for development, as they provide positional information to activate downstream genes and processes in the proper locations. Previous work showed that a haploid model reconstituting the known interactions within this network can robustly reproduce the observed pattern of gene expression (i.e. the phenotype) despite large changes in the model parameters representing the biochemical kinetics [2],[38],[39].

To investigate the evolution of phenotypic robustness of the segment polarity network, we developed a novel approach where model parameters were calculated from a digital genotype, allowing our model to bridge genotype to phenotype (the pattern of gene expression) in a way that can capture the quantitative and qualitative effects of mutation and recombination. Mutations can alter the strength of interactions, and all connections/processes in the network can vary and evolve in a simulated population of organisms. Additionally, we built a diploid model of this network, which allows 2 versions of each gene and all resulting gene products to have potentially different kinetics. Using these, we explore how and whether a diploid model is more robust compared to the haploid. We simulate a population of individuals (organisms endowed with the network), with selection only to stabilize the correct spatiotemporal pattern of expression (phenotype). Using this more biologically detailed representation of the segment polarity gene network we compared evolution of robustness in 4 different populations: sexual haploid, asexual haploid, sexual diploid, and asexual diploid. We find that diploid sexual networks evolve the greatest robustness increase and the combination of the two produces greater robustness than either alone.

## Models

**Figure 1:**
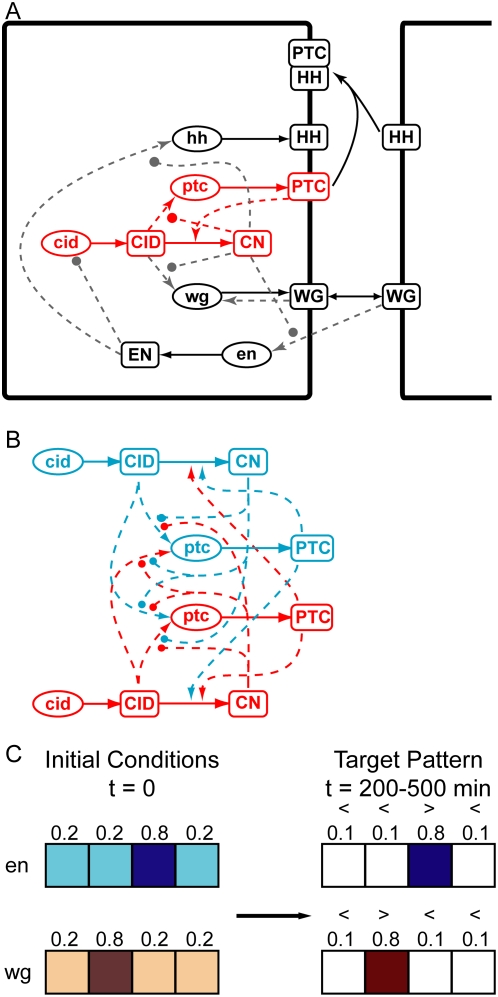
Model of the segment polarity network. (A) Interactions in the haploid segment polarity network adapted from von Dassow (2000). The model incorporates regulatory interactions between 5 genes in the segment polarity network. mRNAs are indicated by lowercase ovals, proteins by uppercase squares. Solid lines indicate fluxes, dashed lines are regulatory interactions, activators end in arrowheads, inhibitors end in circles. Large rectangles indicate cell membranes. The model simulates a row of 4 cells endowed with identical networks to that shown here. The row of cells has toroidal topology and simulates a 2-D sheet of cells. (B) A piece of the diploid segment polarity network showing the subset of interactions drawn red in (A). In the diploid network, each gene has 2 alleles with the corresponding products that participate in the same regulatory interactions but may do so with quantitative differences between the 2 alleles. The number of regulatory interactions in the diploid network can be more than doubled because of the increased combinatorics in diploid networks. (C) Initial conditions for en and wg gene expression(left), and we required the segment polarity network sharpen the en and wg expression by 200 minutes (right). Cells with low initial expression of wg and en must have even lower expression by 200 minutes of development, while the cells expressing initially high wg and en must maintain high expression.

We took as a starting point a previous haploid model of the segment polarity network [2],[38],[40]. This model, shown in Figure 1A, reconstitutes the core biological interactions as a set of ordinary differential equations that govern the time evolution of mRNA and protein concentrations in a row of 4 cells, starting from the prepattern of wg and en mRNA expression shown in Figure 1C. The spatiotemporal pattern of expression depends on the biochemical parameters in the model. Thus, the model is a bridge between a kinetic description of the network and the spatial pattern of gene expression, the phenotype.

**Figure 2:**
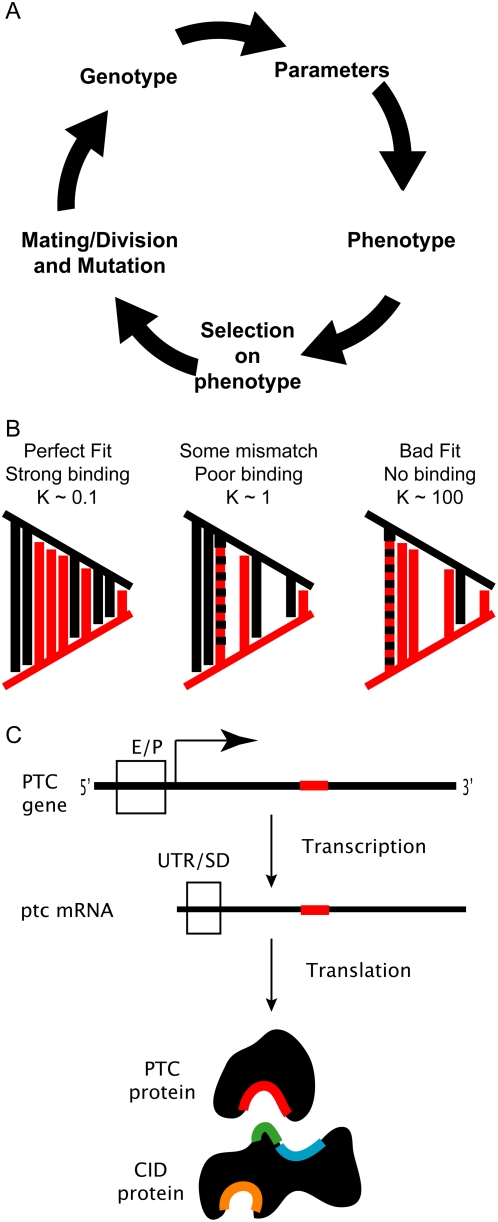
Bridging genotype to phenotype to simulate evolution. (A) Flowchart of evolutionary model. A population of individuals is generated, and their genotype determines their phenotype. Individuals are subject to either truncation or stabilizing selection, and viable individuals mate (if sexual) or divide (if asexual). Each gene in the next generation has a small (3%) chance of having a mutation. (B) Each model parameter was determined from genotype represented as a bit sequence. The model parameter value was calculated from the amount of complementarity between 2 different bit sequences with a length of 20 bits (figure shows 10 bits for simplicity), representing the shapes of interacting surfaces in the biomolecules. Black and red lines are graphical representations of the shapes these bit sequences represent; 1's indicate protrusions, 0's indicate crevices. Each bit is weighted double that to its right, and the strength of the interaction is scaled by the binary exclusive OR (XOR) between the two bit sequences. A perfect fit in a binding interaction would have a low dissociation constant, while worse fits would have corresponding looser binding. (C) Mutations may have specific effects depending on the location in the gene. Each gene had many separate bit sequences, one for each parameter in the model that the gene was involved in, corresponding to the different quantitative effects of mutation. For example, a mutation in the enhancer or promoter sequence (E/P) would alter gene expression levels, while mutations in the 5′ untranslated region or translation initiation site (UTR/SD) would alter translation rates. Mutations in the coding region that alter the binding site for CID on the PTC protein (Red) would alter the ability of PTC to cleave CID. Similarly, the different active sites on the CID protein (green, blue, orange) could be specifically altered by mutations in the coding region.

In the following paragraphs, we describe extensions to this model that allow us to simulate evolution of the segment polarity network in response to selection on the pattern of en and wg expression (the phenotype). We present a diploid version of the model that allows us to directly compare evolution and robustness in haploid and diploid models. We also use a novel framework of deriving model parameter values from a digital genotype, which allows mutations to alter many gene properties (i.e. changes in expression level, stability and activity). Using these, we start with initially viable identical founders and follow them through 2,000 generations of evolution as shown in Figure 2A. We use the model to calculate phenotype (the en and wg pattern of expression) from genotype, apply truncation or stabilizing selection on the phenotype, using a multinomial sampling scheme to simulate random mating with a fixed population size (N = 200) and a per-gene mutation rate (μ) of 0.03.

### Model of the Haploid Segment Polarity Network

Mathematically, our model of the haploid segment polarity network is the same as described previously [2],[38] with 2 modifications: (1) The equations incorporate parameters for transcriptional and translational synthesis rates (which were previously removed by nondimensionalization). Including these parameters does not alter the dynamic repertoire of the system, and allows mutations to alter the expression levels of the mRNA & proteins. (2) Cells were 4-sided (changed from 6-sided) to allow faster computation. This change did not alter the hit rate of successful solutions in a random parameter search, nor did we notice a change in the dynamical behavior of the system.

The segment polarity network was reconstituted into a system of ordinary differential equations. The dependent variables in this system represent the concentrations of the biomolecules in each cell (for cytoplasmic/nuclear molecules) or membrane compartment (for membrane-bound molecules). The system simulates a row (4 cells wide) of square cells with repeating (toroidal) boundary conditions to represent a 2-D sheet of interacting cells. The concentration of a membrane-bound protein can be different on each of the 4 sides of a cell (each side is treated as a separate compartment), and we simulate diffusion by allowing molecules to transfer between cells and membrane compartments where appropriate. The time rate of change for a given concentration is simply the sum of the processes/mechanisms influencing it:
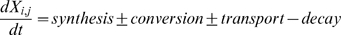
(1)where 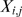 is the concentration of molecule *X* in side *j* of cell *i*.

Decay, binding, and translation follow standard mass-action kinetics (1^st^ or 2^nd^ order). The detailed kinetics of enzymatic activity and translational activation have not been measured in the segment polarity network, so these processes are constructed from Hill functions as described previously [2],[40]. Briefly, if protein A activates production of molecule X, then:
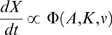
(2)where 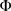 is the Hill function:
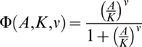
(3)and where *A* is the concentration of the activator, *X* is the concentration of target, *K* is the concentration of *A* where activation is half maximal, and *ν* is the cooperativity (Hill coefficient). This parameterization is attractive because it can be tuned to capture a wide range of activation curves with parameters that are commonly used in standard enzyme kinetics and these parameters are, in principle, measurable. Additionally, this function enforces expected qualitative behavior of biological processes: saturation (biological processes tend to saturate above some level of activation, after which further addition of activator ceases to have an effect) and monotonicity.

The complete list of equations and parameters are listed in Protocol S1 and Tables S1 and S2. All software was written in Mathematica version 5.2 (Wolfram Research). The system of equations was integrated using Mathematica's built-in NDSolve numerical differential equation solver. To guard against errors in numerical integration, we tested a subset of the solutions generated by Mathematica to that returned by Ingeneue [40],[41]. Ingeneue uses a different numerical integration scheme than Mathematica, and shares no code, and we found no difference between the solutions returned by the two programs.

### From Haploid to Diploid Models of the Segment Polarity Network

The model shown in Figure 1A is a haploid network, with a single form of each gene. We constructed a diploid model of the network with 2 versions of each gene and gene product. Figure 1B shows the diploid network for only the *ptc* and *cid* genes; both the number of distinct biomolecules (boxed items) and the number of interactions (lines) can increase by a factor of 2 or more. In the diploid model, there are 2 distinct versions of each mRNA and protein. However, for complexes, such as the Patched-Hedgehog dimer, there are 4 possible distinct dimers (4 ways to combine the 2 HH and 2 PTC proteins).

In the diploid network, all molecules maintain the same activities as in the haploid, but the presence of two alleles must be correctly implemented to follow the established biology of diploidy. Fluxes/conversions (solid lines in the network diagram) are doublings of the haploid version: translation, decay, exo & endocyctosis, and diffusion. For example, each protein is translated only from the corresponding mRNA; i.e. CID1 protein is translated only from cid1 mRNA, while CID2 protein is translated from cid2 mRNA (and we assume it is independent of cid1 translation). Similarly, the two versions of each biomolecule decay independently with 1^st^ order kinetics (we assume the decay of one allele does not affect the rate of decay of its homologue). Regulatory interactions (dotted lines in the network) become more complex in the diploid network, as we must account for the combined regulatory activity of both alleles. In the example in Figure 1B, each of the two CID proteins can have a potentially different effect on the activity of each of the ptc target genes, so the number of arrows (regulatory interactions) has quadrupled compared to the haploid case.

**Figure 3:**
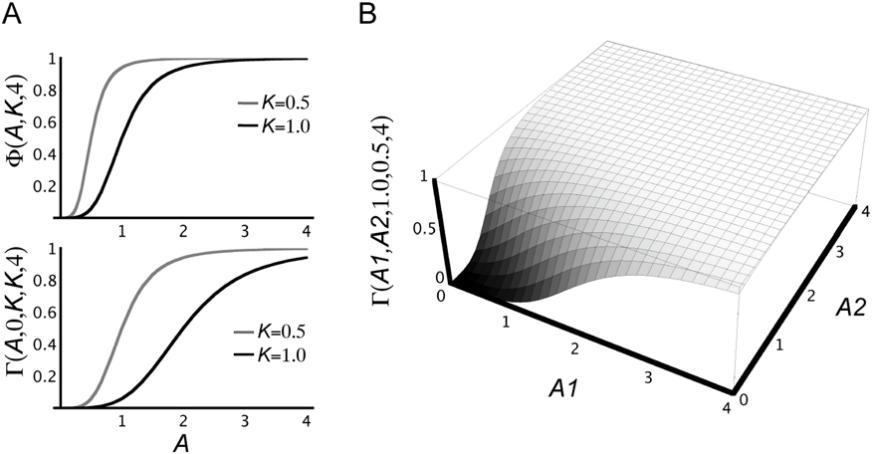
Activation functions for transcription control were constructed from Hill-like functions. (A) Activation curves for haploid or homozygous diploid (upper graph) and diploid with complete loss of one activator (lower graph). Upper graph plots Equation 3. For a homozygous diploid model, we constructed the activation curves to reduce to be identical to the haploid (see Equations 3 & 5). Lower graph plots Equation 5 following the complete loss of one activator. In this case, the *K* parameters are no longer half maximal activations, but still lower values correspond to stronger action. (B) The general behavior of diploid activation by two activator alleles of different strength, according to Equation 5. In our model, the loss of one allele can be compensated by a sufficiently large increase in another allele.

For diploid networks, we construct an extension of the Hill function to allow for two activators controlling expression of a target. Here, we extend the example of Equation 2 for two activators (A1 and A2) that can have different efficacies in activating two targets (X1 and X2):
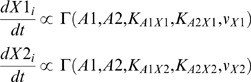
(4)where *X*1 and *X*2 are the concentrations of the two alleles of target gene X, and *A*1 and *A*2 are the concentrations of the two alleles of activator A, and 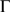 is an extension of 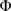 (described below). *K_A_*_1*X*1_ describes how efficiently *A*1 influences *X*1 synthesis (i.e. how well transcription factor A1 activates the production synthesis of X1 by binding productively to the X1 enhancer sequence), *K_A2X_*_1_ describes how efficiently *A*2 influences *X*1 synthesis(i.e. how well transcription factor A2 activates the production synthesis of X1 by binding productively to the X1 enhancer sequence), etc. We assume that A1 and A2 proteins do not interact with each other in activating X (i.e. we do not consider that A1 might block activity of A2 by nonproductively binding to the enhancer sites on X1) and the net activity of A1 and A2 is simply their average activity in binding to the affector for gene X. Furthermore, we assume the cooperativity reflects the number of occupied binding sites on the target gene. Substituting 
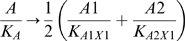
 into the Hill equation yields:
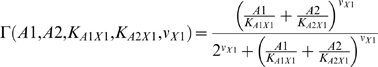
(5)The *K* parameters (*K_A_*_1*X*1_, *K_A_*_2*X*1_, etc.) in the diploid model cannot strictly be interpreted as half maximal activities like their haploid counterparts because activation depends on both *A1* and *A2*. Note that a completely homozygous diploid is identical to the haploid; when the concentrations of both diploid activators and activities are the same (when *A* = *A*1 = *A*2 and *K* = *K*_1_ = *K*_2_) then 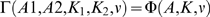. Figure 3 shows the behavior of Equations 3 and 5.

There are many ways to extend the Hill function (or implement alternative formulations) to approximate the effects of diploidy, and increased realism comes at additional computational complexity. A highly realistic model would ideally track the bound state of each enhancer site for a gene (perhaps including current availability of the site based on histone acetylation, etc.), the affinity of each activator allele for each site, and the contribution of each bound transcription factor to the initiation of transcription. We settled upon the formulation in Equation 5 because it is simple (both to use and understand) but captures attractive features of diploidy. Specifically, our scheme: (1) Captures the same qualitative biological behavior as the Hill function did in the haploid case: saturation and monotonicity. (2) Does not dramatically increase the parameter count or complexity of the model. (3) Allows for direct comparison between the haploid and diploid models. The homozygous diploid model reduces to the haploid equivalent when *A*1 = *A*2 and *K*_1_ = *K*_2_. This allows us to compare directly the evolution of diploid and haploid networks.

Our formulation of Equation 5 has consequences for the behavior of heterozygous diploid networks. Activation in the diploid model depends on the average activity (concentration divided by *K* parameter) of the two activators. Thus, the loss of either A1 or A2 can be compensated by a sufficiently large increase in the concentration of the other (shown in Figure 3). In the case of a homozygous diploid, if *A1* = *A2* and both have the same activity (both have identical *K*'s), then the total activity is the same as the haploid. Depending on the activities of the two activator proteins A1 and A2, the loss of either could result in anything from an insignificant change (if *A1* and *A2* were both far above their respective *K*'s) to a dramatic change (if *A1* and *A2* were near their respective *K*'s).

We emphasize that the segment polarity network has highly nonlinear behavior [2],[38], and the loss of one allele in an otherwise homozygous individual will usually not result in a simple halving of expression in the affected gene. There is substantial feedback between different genes and different cells, and some perturbations can result in a complete change in the pattern of expression, while others will produce almost no change. Because there are multiple cells in the network that are co-regulating each other, many genes must be expressed within a correct window of expression (above one threshold but simultaneously below another) in each of the cells. Additionally, when there is high cooperativity in Equations 3 and 5, the resulting gene activity may be unchanged (if far from the threshold for activity) or completely lost (if near threshold).

Our implementation of diploidy does not allow for the possibility of interactions between the two activator alleles: for example that A1 and A2 compete for binding sites in such a way that A1 fails to activate production of X and also (dominantly) blocks the activity of A2 (by binding nonproductively to enhancer sites). Similarly, we do not allow for overdominance effects such as A1 and A2 somehow synergizing so that their combination has greater activity than an equivalent amount of either alone.

### Representing Genotype

The models of the segment polarity network described above are insufficient to predict the effects of mutations on phenotype because many parameters in the model are not properties of individual gene products, but instead reflect *interactions* between biomolecules. For example, many parameters in our model determine how well a transcription factor activates or inhibits its target's gene expression. In reality, the strength of such regulatory interactions could be altered by mutating either the transcription factor or enhancer sequence, resulting in different patterns of inheritance depending which gene combination is passed on to the offspring. Additionally, a single mutation can perturb multiple parameters in the model: a mutation in a transcription factor will affect its ability to recognize both enhancer sequences.

Biophysically, interactions in genetic networks rely on physical binding of biomolecules in regions with complementary surface chemistry and topology. To capture the qualitative behavior of such binding, we abstract genotype as a bit sequence (digital genotype) comprised of 1's & 0's that can be imagined as a surrogate for the physical surface of molecules that participate in a binding interaction (i.e. an enzyme's active site or the binding surface offered by an enhancer consensus sequence) as shown in Figure 2B. The strength/kinetics of an interaction (represented by biochemical parameters in our model) are determined by the degree of complementarity between two bit sequences, weighted by bit position. Each bit in the sequence is weighted twice that of its neighbor on the right to allow mutations that alter bit-sequences to have graded effects from very small to large (the motivation for this choice is further discussed in the section “simulating mutation” below). The parameter is derived from the interacting bit sequences by simply scaling the normalized bitwise XOR value of the bit sequences according to either a linear
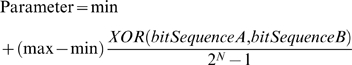
(6)or logarithmic scaling:
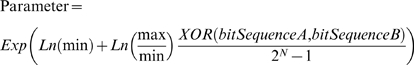
(7)
*bitSequenceA* and *bitSequenceB* are the numeric representations of the binary interacting sequences and *N* is the length of the bit sequence, set to 20 for our simulations. We used linear scaling (Equation 6) for *K* parameters, and logarithmic scaling (Equation 7) for all others. Linear vs. log scaling was used so that mutations usually resulted in a weak/nonexistent interaction as described in the “simulating mutation” section below. Cooperativities were restricted to integer values by rounding the results of Equation 6 to the nearest integer in order to speed numerical integration.

Several parameters reflect the interaction of the segment polarity genes with genes products outside of the network. Table S1 lists the general categories of parameters in the model, what they represent, and indicates whether the parameter is derived from two different bit sequences (i.e. is an interaction between 2 genes with the segment polarity network) or is derived from the comparison of a bit sequence from a single gene with 0 (indicating interaction with general cellular machinery that we assume is constant). For example, maximal transcription and translation rates (*C* and *L* parameters) are determined by how well the SPN genes interact with the initiation machinery for these processes. Evolution of global cellular behavior is slow, while transcription factors evolve quickly [42], therefore we did not allow global machinery to change, and held the corresponding bit sequences fixed at 0 (i.e. all 0's in the bit sequence, this was chosen for convenience since again, this sequence did not evolve). This allows the maximum translation rates of genes to be changed and inherited as any other property, but does not allow, for example, heritable ribosomal mutations that would globally alter all translation rates. Thus, our model explicitly represents the genotype of 5 genes in the haploid network (10 in the diploid).

For the special case of the lifetime of the PTC-HH protein dimer(*H_PH_*), we reasoned that the stability of the complex reflects a tripartite interaction involving both proteins with the degradation machinery in the cell: 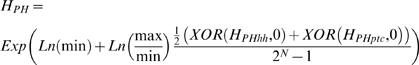
(8)Where min and max are the range of allowed values (Table S2), *H_PHptc_* is the bit sequence representing the ability of the PTC part of the PTC HH dimer to interact with the degradation machinery, and *H_PHhh_* represents the same for the HH part of the dimer. The lifetime of the dimer is the average of the contribution of the ability of HH to be recognized by the degradation machinery (bit sequence fixed at 0) and the PTC part.

In the model, different parameters for each gene describe distinct sub-activities/properties such as mRNA stability, protein stability, protein activity, expression level, etc., as shown in Figure 2C. In reality, the DNA sequences determining these different activities are usually spatially separated on the gene: enhancer sites (affecting transcription rate) are on the non-coding region usually away from the ribosomal recognition sequence (which affects translation rate) and likewise distant from the coding region of the active site (which affects protein activity). Thus, most point mutations alter only one or a few properties of the gene products: for example a mutation in the coding sequence for a real protein might alter the protein's activity and stability [43], but not its transcription rate. Additionally, mutations in a transcription factor can alter its interactions with a subset of targets while leaving other interactions unaffected [44]. To capture this in our model, *we use a separate bit sequence for each of a gene's parameters (sub-activities)*. For example, we use separate bit sequences for the maximum transcription rate of a gene, the stability (mean lifetime) of the mRNA, maximum translation rate into protein, stability of the protein, and each of the protein's activities. Thus, though there are 5 genes (10 in the diploid model), there are far more bit sequences (∼71 in the haploid model, 142 in the diploid) than genes. From these bit sequences, all model parameters (57 haploid, 140 diploid) are determined using Equations 6–8. The number of model parameters is more than half the number of bit sequences because several parameters are derived by comparing bit sequences describing properties of segment polarity genes with fixed cellular machinery (fixed at a value of 0, and not included in the bit sequence count). Thus, parameters that reflect interaction with cellular machinery are simply inherited (though they can still be mutated).

Equations 6 & 7 capture important relationships between different parameters in the model. In a diploid organism, consider a mutation in a transcription factor that affects the surface of the transcription factor that binds the enhancer. Such a mutation will alter the ability of the transcription factor to recognize the enhancer sequences of both target alleles: in Equation 4, a mutation that alters the ability of transcription factor A1 to bind to enhancer sites will alter both *K_A1X1_* and *K_A1X2_*. Conversely, a mutation in an enhancer sequence will alter the ability of both transcription factor alleles to regulate the mutated gene. In Equation 4, a mutation that alters the enhancer sequence of X1 will alter the ability of both transcription factors, A1 and A2, to recognize it and will alter both *K_A1X1_* and *K_A2X1_*. If bit sequence *B_A1 transcription_* represents the surface of A1 that binds to enhancers, *B_A2 transcription_* represents the surface of A2 that binds to enhancers, *B_X1 enhancer_* is the surface presented by gene X1 recognizable by transcription factors, and *B_X2 enhancer_* is the surface presented by gene X2 to transcription factors, then we can calculate the relative strengths of the two transcription factors to activate each target gene using Equation 6:
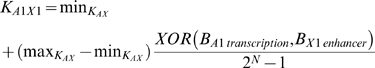
(9a)

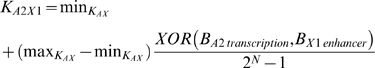
(9b)

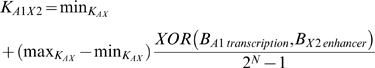
(9c)

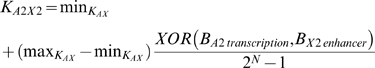
(9d)All 4 *K_AX_* parameters share a common range from 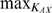 to 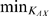. A single mutated bit sequence can affect multiple parameters, as expected from the underlying biology, and our model properly captures the qualitative effects of *cis* and *trans* mutations.

To reiterate, our scheme of calculating parameters from Equations 6–8 is attractive because: (1) It is conceptually consistent with the underlying biophysical mechanism of binding. The binding surfaces/active sites are specified either directly by the genotype (i.e. a regulatory consensus sequence) or indirectly (the genotype specifies the 3-D shape of a protein), but the ultimate origin of both is a mutable sequence (the DNA sequence of the gene). (2) It allows us to compute how well a gene product can interact with any partner, allowing us to easily simulate the effects of recombination (which will produce new combinations of alleles that may not have worked together before) and inheritance, as parameter values are interactions (not heritable) that depend on the interacting genes. Our scheme allows us to calculate the strength of an interaction when, for example, both a transcription factor and the enhancer sequence it binds are mutated. (3) It allows us to simulate both *cis* and *trans* mutations. Transcriptional regulation can be altered by a mutation in either the transcription factor or the enhancer, with different consequences depending on which is mutated. Our bit sequence representation allows this aspect of biological reality to be captured. (4) It allows us to capture the general qualitative features of mutations (see next section). (5) It is computationally trivial.

### Generation of Founder Genotype

To simulate evolution of the network, it was necessary to generate founder genotypes that produced a viable phenotype (Figure 1C). To do this, we performed a random search for viable haploid parameter sets, then converted them to genotypes. To reduce the number of free parameters in the random parameter search, we restricted the transcriptional and translational rates (*C* and *L* parameters) to the inverse of the mRNA and protein lifetimes (*H* parameters):
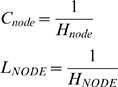
(10)This is equivalent to the nondimensionalization scheme used previously [2],[40]. This strategy was used for the en, wg, ptc, and cid mRNA and proteins. However, because the HH protein will heterodimerize with PTC protein on adjacent cells, we allowed for a stoichiometric excess/scarcity of PTC and HH. In the random parameter search the *L_HH_* parameter varied from 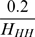 to 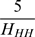. This allows the maximal HH protein concentration to vary from 0.2 to 5 times that of PTC. The restriction in Equation 10 was not applied during evolutionary simulation (i.e. synthesis and stability were independent).

Table S2 shows the range explored for each parameter in the random search for founders. The constraints we impose in Equation 10 enforce that en and wg have a maximal value of 1, and so all founders have similar patterns of wg and en: in cells that should express them highly, wg and en are expressed between 0.8 and 1 during the relevant simulation time from 200–500 minutes. As shown in Table S2, during evolution we allow model parameters to explore a much larger range for most parameters, so wg and en expression can rise above the founder levels to a maximum of 20.

To generate the founder genotypes, we converted working parameter sets into the corresponding genotypes by inverting Equations 6 & 7. The parameter value uniquely defines only the XOR difference between pairs of bit sequences. Thus, we chose a random value for one bit sequence (each bit position was randomly set to 0 or 1 with equal probability), then assign a unique value to the other using the inverse of Equations 6 and 7 above:
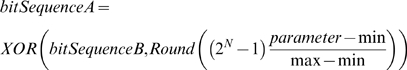
(11)for linearly scaled parameters or
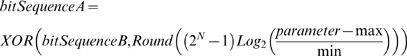
(12)for logarithmically scaled parameters. In Equations 11 & 12, *bitSequenceB* has each position randomly set to 1 or 0, allowing us to find a unique value for *bitSequenceA*. Here, min and max represent the extremes of the allowed values for the parameter during evolution according to Table S2. During evolution, we allow a much wider range of parameter values than during the search for founders, as mutations should often weaken (but rarely strengthen) an interaction.

### Simulating Mutation

We emphasize that the bit sequences described in the previous section are abstract surrogates for the 3-D physical surfaces of molecules that participate in an interaction. They do not attempt to represent base pairing between complementary nucleotide sequences as all interactions in the segment polarity network are protein–protein or DNA/RNA-protein interactions. There is no general theory that allows us to calculate the strength of binding between an arbitrary gene product and its partners, nor to predict the effect of a general mutation on the strength of this binding. Quantitatively, a point mutation can have varied effects on an interaction: a complete quenching of an interaction (mutation of the nucleotide/amino acid that is essential for binding/interaction), an almost imperceptible change (mutation at a site peripheral to the key interaction that slightly perturbs the interaction strength), a (less likely) strengthening of the interaction (a mutation that slightly increases the affinity of binding). In general, most mutations lower the expression and activity of gene products, though rare mutations may strengthen them.

After each mating/division in our evolutionary simulation, we allow a 3% chance per gene of a mutation. In a mutated gene, we mutate a randomly-chosen bit sequence, with a recursive 10% chance that an additional bit randomly-chosen bit sequence in the same gene is mutated. Thus, mutations typically change one bit sequence (90% probability per mutation) or more than one (10% probability per mutation) in the gene, allowing mutations to, for example, change both the activity and stability of a gene product. When a bit sequence is mutated, we randomize each position of the bit sequence to a 0 or 1 (mutations result in an independent random draw). The effect of this is the corresponding parameter(s) is/are set to a value between the min and max shown in Table S2. For example, the mean lifetime for a gene product (*H* parameters) will have a log-distributed random value between 1×10^−6^ and 100 after a mutation (mean = 1×10^−2^; a factor of 500 lower than the most unstable founder). Thus, 75% of mutations will result in near to complete elimination of a gene product (with a mean lifetime less than 1; in the founders, mean lifetimes vary from 5–100). Only a fraction (∼12%) of mutations will produce protein stabilities comparable to those of the founders. Similarly, values for transcriptional regulation (half maximal concentrations or *K* parameters) will have a random value uniformly distributed between 0.001 and 100 (mean = 50, a factor of 100 higher than most founders and no gene product in any simulation evolved expression high enough to activate a process with such a weak interaction). This biases the parameter towards extremely high values (i.e. weak activity), with >99% of mutations producing ineffective (or dramatically lowered) transcriptional regulation. For the special case of cooperativities (*ν* parameters), we restricted these values to a narrow range (mutations produce integer cooperativities between 1 and 10), as high cooperativities are computationally expensive. Thus mutations usually produce a limited change in cooperativity, biased towards low cooperativity (log scaled). For all other parameters (>80% of parameters), mutations, on average, produce interactions 2+ orders of magnitude weaker than the founders.

In our model, mutations usually result in very weak or absent interactions (i.e. the corresponding parameter has a value so the interaction is silent). We have not attempted to reproduce the real distribution of mutational effects. Our model parameters abstract a wide variety of processes (RNA stabilities, protein stabilities, transfer/diffusion rates, etc.). For many processes, the mutational effects are not well known, and capturing the remaining known mutational spectra would require a separate mutational scheme (or genotype→parameter mapping function or both) for each class of parameter. Our goal was to allow mutations to have graded effects that usually disrupt interactions but occasionally strengthen them, and also allow us to calculate the strength of interaction between arbitrary pairs of partners (that may not have co-existed within the same individual before). Additional limitations of our mutation scheme: (1) We do not allow the possibility of whole gene duplications or genes to evolve novel interactions that are absent from Figure 1A (i.e. dimerization between en and wg or PTC degrading en). (2) We do not attempt to capture the relative rates or magnitudes of mutational effects: one could imagine that mutations may more frequently alter a protein's mean lifetime than the per-molecule maximal catalytic rate due to the differences in mutational target size. Similarly, the magnitude of mutational effects may differ: individual amino acids may contribute weakly to the overall protein stability while mutations in the active site may dramatically alter catalytic rate.

### Simulating Mating and Recombination

In sexual populations, mating was random, with randomly chosen (with replacement) pairs of parents producing a single offspring. Recombination proceeded as follows: In diploid sexual populations, each parent would randomly pass on one of its two alleles for each gene to the offspring (we did not include the effects of genetic linkage in this study). In haploid sexual populations, the haploid offspring produced by mating two haploid parents would randomly inherit (with 50% chance) one of the two parents' alleles for each gene. In both cases, all bit sequences corresponding to an inherited gene were passed on together, and we did not allow recombination within genes. Division in asexual populations was implemented by allowing a randomly chosen individual to produce a clonal offspring that had the same genotype as the parent. In all simulations (sexual and asexual), the genotype was subject to mutation as described above, and individuals reproduced until the specified number of viable offspring reached the population limit. Drift is present in our simulations, as an unlucky individual may stochastically not mate/produce any offspring, and individuals could mate with more than one partner in each generation.

### Simulating Evolution

We began by screening many randomly generated haploid genotypes to find 40 “founder” genotypes that sharpened the pattern of wg and en mRNA expression as shown in Figure 1C. We simulated evolution for 2,000 generations starting each simulation with a single founder. For each founder, we simulated 4 independent parallel runs: sexual haploids, asexual haploids, sexual diploids, and asexual diploids. Forty diploid founder genotypes were constructed from the haploid founders by making them homozygous for the haploid alleles (again, diploids homozygous for all genes produce the identical phenotype as the haploid). Each generation in our model of evolution comprised the 5 phases shown in Figure 2A: Prediction of model parameters from genotype, determining phenotype (spatiotemporal pattern of wg and en expression), selection on phenotype, reproduction (either sexual or asexual cloning), and mutation. Population size was fixed at N = 200, giving 100 (diploids) or 200 (haploids) in each generation.

We used one of two selection criteria in our simulations: stabilizing or truncation. Genomic data suggests that gene expression in *Drosophila* is under stabilizing selection [45]–[47], or selection for an unchanging pattern of expression. In our stabilizing selection simulations, the founder phenotype is optimal (fitness 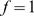), with fitness falling as the en and wg patterns diverge from the founder phenotype. Quantitatively, fitness 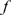 under stabilizing selection is: 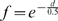
(13)where *d* is the phenotypic distance between the (optimal) founder and the evolved individual. For haploid individuals:
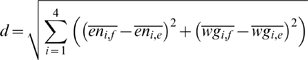
(14)and for diploid individuals where there are 2 potentially distinct en and wg alleles:
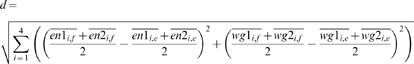
(15)where *en_i,e_* and *wg_i,e_* are the en and wg mRNA concentrations in the *i* ^th^ cell position in an individual whose fitness is being determined, *en_i,f_* and *wg_i,f_* are the levels of en and wg expression of the (optimal) founder, and horizontal lines indicate time averages of the concentration from 200 to 500 minutes of development. When diploids are homozygous for all alleles (producing identical expression of both en and wg alleles), *d* reduces to the haploid case.

The developmental function of the segment polarity network is to stabilize stripes of gene expression to pattern subsequent development. From the perspective of this function, mutations that produce insufficiently sharpened wg and en stripes are disastrous while those that result in an over sharpened pattern are viable. To explore the consequences of this, we simulated truncation selection where individuals are dead (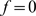) if wg and en have expression levels outside of the expression thresholds shown in Figure 1C, or take too long to stabilize their correct patterns. Otherwise, individuals are viable with 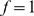. In other words, as long as en and wg are sufficiently high in the correct cells (and sufficiently low in the rest), the developmental processes that depend on wg and en expression are unperturbed and the individual will be viable. These two criteria approximate two biologically plausible extremes, truncation selection penalizing insufficient sharpening of the pattern but allowing the pattern to change, while stabilizing selection penalizes any deviation from the founder pattern.

### Measurement of Robustness

We tested robustness to 3 types of perturbations that the real segment polarity network might be exposed to: (1) Perturbation of a single bit sequence in a single randomly chosen gene. This usually caused a dramatic change in one or two parameters, and is conceptually similar to a point mutation that produces a specific effect. (2) Perturbation of all parameters. We multiplied each parameter (after calculating it from genotype) by a randomly-chosen (log-sampled) value from 0.66 to 1.5, independently (i.e. all parameters were perturbed by a factor up to 1.5). Extreme environmental stress (pH change, temperature change, starvation, etc.) could alter the cellular environment so many parameters are substantially altered. (3) Perturbation of initial conditions. We multiplied the initial amount of wg and en mRNA by a randomly-chosen (log-sampled) value from 0.5 to 2, independently in each cell (i.e. noise was added to the en and wg prepattern, but this never changed the positions of the cells with the highest initial en and wg). A variety of sources (developmental noise, mutations in genes responsible for the en and wg prepattern, etc) could result in a perturbed prepattern.

We quantified robustness to these sources of variability and, for clarity, we will use the term ‘survivorship’ when describing results from truncation selection and ‘fitness’ for stabilizing selection. Under truncation selection, we measured the fraction of trials where the ability to sharpen the pre-pattern (according to the criteria in Figure 1C) continued in the face of perturbation. Under stabilizing selection we measured the fitness decrease (Equation 13) using the distance between the unperturbed and perturbed wg and en expression levels analogously to Equations 14 and 15.

## Results

### Diploidy Confers a Robustness Advantage in Random Genotypes

**Figure 4:**
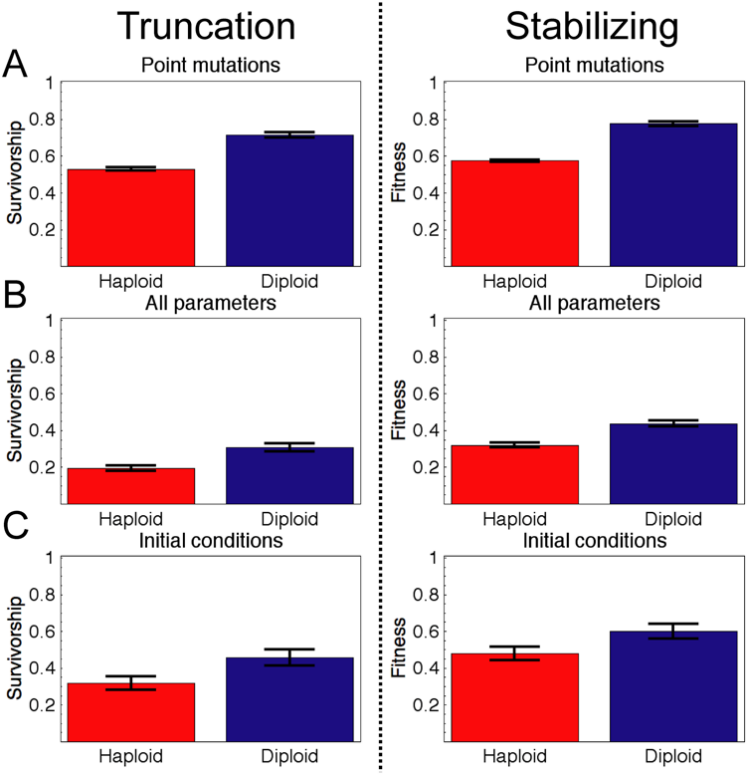
Effect of ploidy on robustness of randomly generated genotypes. Viable haploid (red) and identical homozygous diploid (blue) genotypes were subjected to perturbation. We measured robustness under truncation selection (left column) by measuring the fraction of perturbed individuals that continue to reproduce the threshold pattern shown in Figure 1C. For stabilizing selection(right column) we measured the fitness of the perturbed individuals according to Equation 13. (A) Robustness to a point mutation simulated by randomizing a single bit sequence in a randomly chosen gene. (B) Robustness to independently perturbing all parameters by a factor up to 1.5. (C) Robustness perturbing initial conditions of en and wg in all cells by a factor up to 2. Error bars are standard error of the mean (n = 40 genotypes).

As described in Models, we generated 40 viable random haploid genotypes that stabilized and sharpened the pre-pattern to produce the phenotype shown in Figure 1C. These genotypes were not the product of evolution, but of randomly searching for genotypes satisfying the above criteria. We then measured how robustly the phenotype persisted in the face of perturbation (see Models), comparing the randomly generated haploid genotypes with homozygous diploid genotypes (homozygous for the haploid genotype for all genes). Figure 4 shows the robustness of the diploid and haploid networks. Homozygous diploid networks were substantially more robust to perturbations than their haploid equivalents: diploids had a higher chance to maintain the wg and en sharpening and showed a smaller change in their en and wg patterns compared to their haploid equivalents. The diploid robustness advantage varied with the specific genotype we tested, but diploids had greater robustness than haploids in >90% of the genotypes.

### Diploidy and Sex Allow Evolution of Increased Robustness

We next tested whether the greater robustness of diploid networks persisted when we simulated evolution for 2,000 generations using the same 40 randomly generated, viable genotypes as founders. In these simulations, we used a high mutation rate (μ = 0.03) with small population sizes (N = 200). We used each genotype to generate a genetically identical founder population and simulate evolution under either truncation or stabilizing selection with the following conditions: haploid sexual, haploid asexual, diploid sexual and diploid asexual. Thus, each founder was used in 8 parallel simulations. Our simulations allow us to incorporate key features of diploidy: (1) Genotype is the product of evolution, not from a random search of genotypes that happen to produce the right pattern. (2) There is usually genetic diversity in a population [9], [11]–[13], [48]–[50] and diploid individuals can be heterozygous at loci. (3) Diploid individuals experience twice as many mutations as haploids during evolution (assuming a constant per-gene mutation rate).

Regardless of ploidy or reproduction mode (sexual or asexual), our evolutionary simulations quickly produced a genetically diverse population, with several quantitatively different alleles co-existing for most genes in any given generation (expected since the expected number of mutations per gene per generation μN = 6). Initially, populations were genetically identical at all loci, but the founder allele became extinct within a few hundred generations, after which there was a diversity of several alleles present in the population, and diploid individuals were heterozygous for most genes.

**Figure 5:**
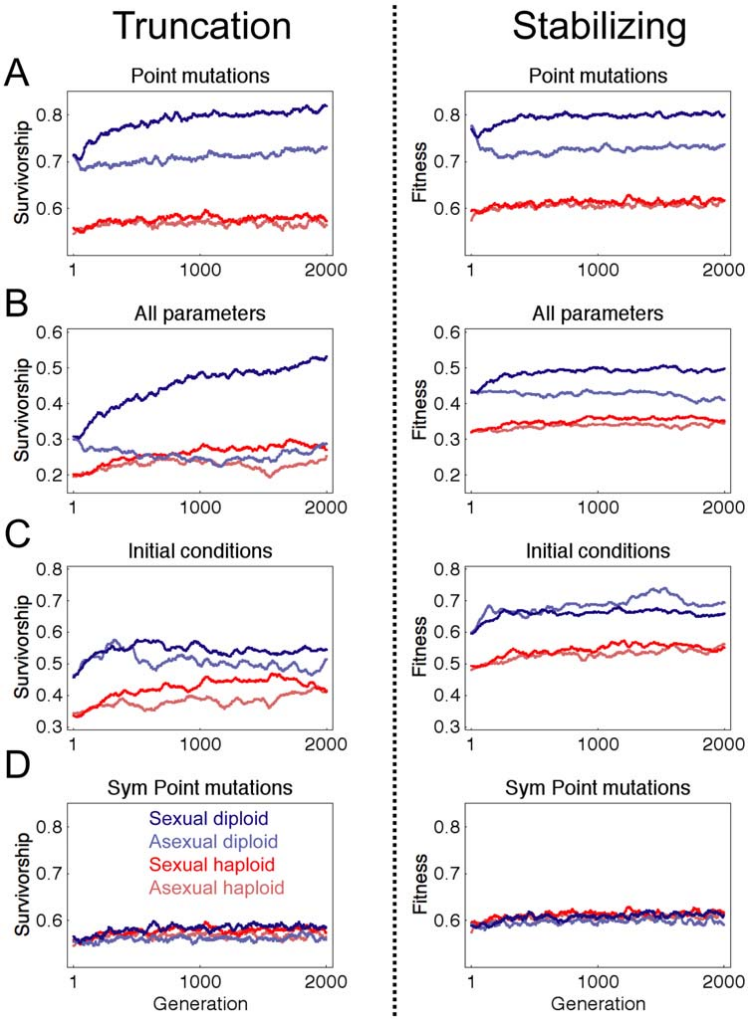
Measurements of robustness in evolving populations. Left column plots truncation selection, right column plots stabilizing selection. Diploid sexual populations evolved the greatest robustness by 2,000 generations regardless of selection criteria. (A–C) Robustness is measured under the same conditions as shown in Figure 4. (D) Symmetric double mutations that perturbed a single property of both alleles in the diploid network identically (see text); haploid plots are unchanged from left column and are shown for comparison. Plots show average of 40 simulations, smoothed with a sliding window over 50 generations.

After simulations were complete, we measured the robustness at each generation to 3 types of perturbation; results are shown in Figure 5A–C for the average of all 40 simulations in each condition. Simulations under truncation and stabilizing selection showed the same qualitative behavior. All populations evolved increased robustness to the perturbations. Diploid populations continued to exhibit increased robustness compared to haploid populations, especially when combined with sexual reproduction. Comparing the terminal generations that share a common founder, diploid sexual populations evolved the greatest robustness at generation 2,000 in almost all (38/40 truncation; 39/40 stabilizing) tests of robustness to point mutations, most (32/40 truncation; 31/40 stabilizing) tests of robustness to all parameter perturbations, and a substantial fraction (19/40 truncation; 18/40 stabilizing) of tests of robustness to initial conditions. While we cannot determine whether the robustness advantages of diploid sexual populations persist forever (i.e. the asymptotic behavior), extrapolating from data in Figure 5 suggests that diploid sexual populations should maintain higher robustness than other conditions far into the future.

### Most Mutations Are Recessive in Diploid Networks

The data shown in Figures 4A and 5A suggest that most mutations in the diploid network model are recessive: simulated point mutations had a smaller effect in diploids than haploids. This is not built in; our network allows for the possibility of dominant deleterious mutations. Examples of possible dominant (and lethal) mutations that we observed during simulated evolution: (1) A sufficiently hyperactive WG protein (which is initially expressed at non-zero levels in all cells in our simulation) could disrupt the normal gene expression pattern through excessive global wg autoactivation or global en activation. (2) A mutation in the enhancer of cid that causes loss of inhibition by en would result in overexpression of CID that disrupts the wg and en patterns. In our simulations, mutations usually result in nonproductive interactions, so mutation (1) is far less likely than mutation (2); the former requires the (unlikely) mutation that produces strong autoactivation while the latter requires a (more frequent) loss-of-function.

There are two mechanisms that may contribute to the increased robustness in diploid populations: First, diploidy allows masking of a perturbed allele by its homologue (i.e. most mutations are recessive). Second, diploid populations may evolve increased robustness faster than their haploid counterparts through a mechanism independent of dominance. To separate these, we measured robustness in diploid populations by simulating symmetric mutations that perturb both versions of an allele by the same amount, so there is no unperturbed homologue to mask the perturbed allele. Symmetric point mutations altered the same bit sequence in both alleles of the perturbed gene by the same amount (both homologous bit sequences were altered by an XOR operation with the same random value). Figure 5D shows the results of symmetrically perturbed diploid populations compared to their singly-perturbed haploid counterparts. The robustness of the symmetrically perturbed diploid populations was very close to the haploids, and changed only slightly over time, indicating that the majority of mutations are recessive in our diploid model of the segment polarity network.

The ability of the network to mask the effects of mutation may itself be evolving (i.e. over time, the network evolves so that more mutations are recessive). Such evolution would manifest in diploid populations as an increase in robustness to (single) point mutation without an increase in robustness to symmetric point mutations. Our data indicates this is the case for diploid sexual populations, as the dramatic increase in robustness to point mutations over time is almost eliminated under symmetric mutation. Diploid asexuals show a far smaller increase, indicating that sex accelerates evolution of greater masking (i.e. greater dominance of functional alleles).

### Diploid Sexual Populations Select Strongly for Compatible Alleles

**Figure 6:**
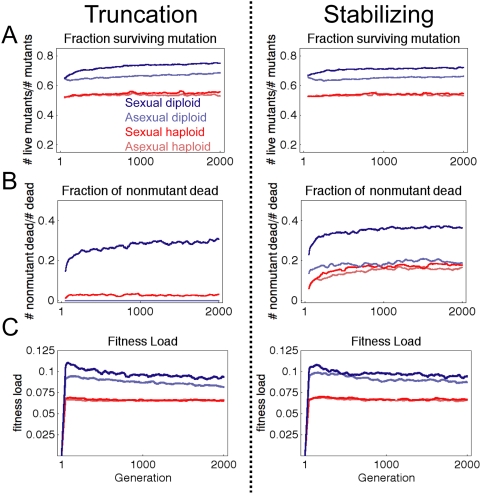
Distinguishing effects of mutation from recombination. (A) Fraction of mutated individuals that were viable during evolutionary simulation. (B) Fraction of dead individuals during the simulation that did not have a mutation. A dramatically higher fraction of deaths were caused by recombination in sexual diploid populations than sexual haploid. (C) Fitness load calculated from Equation 16. Plots show average of 40 simulations, smoothed with a sliding window over 50 generations.

Why does sex produce more robust populations? In our simulations, individuals have reduced fitness/survivorship if they fail to sharpen the correct en and wg patterns sufficiently. Fitness/survivorship can be reduced by two sources: a new mutation or, in sexual populations, recombination of alleles that do not function properly together. Figure 6 shows the relative effect of recombination and mutation on survival. During the simulation, we recorded the number of dead individuals and their genotypes, and whether they had a new mutation. Figure 6A shows the fraction of individuals with a new mutation that were viable. This data is qualitatively consistent with Figure 5A, but includes mutations that could alter multiple genes and bit-sequences during evolution. To determine how often recombination produced incompatible allele combinations, we measured the fraction of deaths where individuals did not have a new mutation (i.e. the fraction of the dead due to recombination). Figure 6B shows diploid sexual populations showed a near doubling of this fraction compared to the haploid sexual populations. Thus, diploid sexual populations experience a greater pressure to maintain alleles that both produce the correct phenotype and that are also highly compatible with the other alleles in the population. Recombination constantly produces new allele combinations that cause quantitative variation; thus sexual populations (especially diploid sexual populations) more strongly select for genotypes (and alleles) that are robust to quantitative variation.

In Figure 6C, we plot the fitness load for each of our simulations defined as:
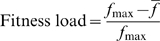
(16)where 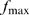 is the fitness of the most fit individual in the generation, and 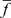 is the mean fitness of all individuals in that generation. Consistent with Figure 6B, we see that recombination produces a higher fitness load in diploid populations (the fitness load is noticeably higher in diploid sexual populations compared to diploid asexuals), but not haploid populations (the fitness load of the two haploid populations are nearly equal). Proulx and Phillips [14] showed the upper bound for selection for mutational robustness is the fitness load minus the mutation rate. All 4 populations have fitness loads higher than μ, with diploid sexual populations having the greatest expected pressure to evolve (and maintain) mutational robustness. Taken together, our data shows that the combination of sex with diploidy synergize to produce the strongest selection for mutational robustness.

### Phenotypes Move Away from Some Selection Thresholds

**Figure 7:**
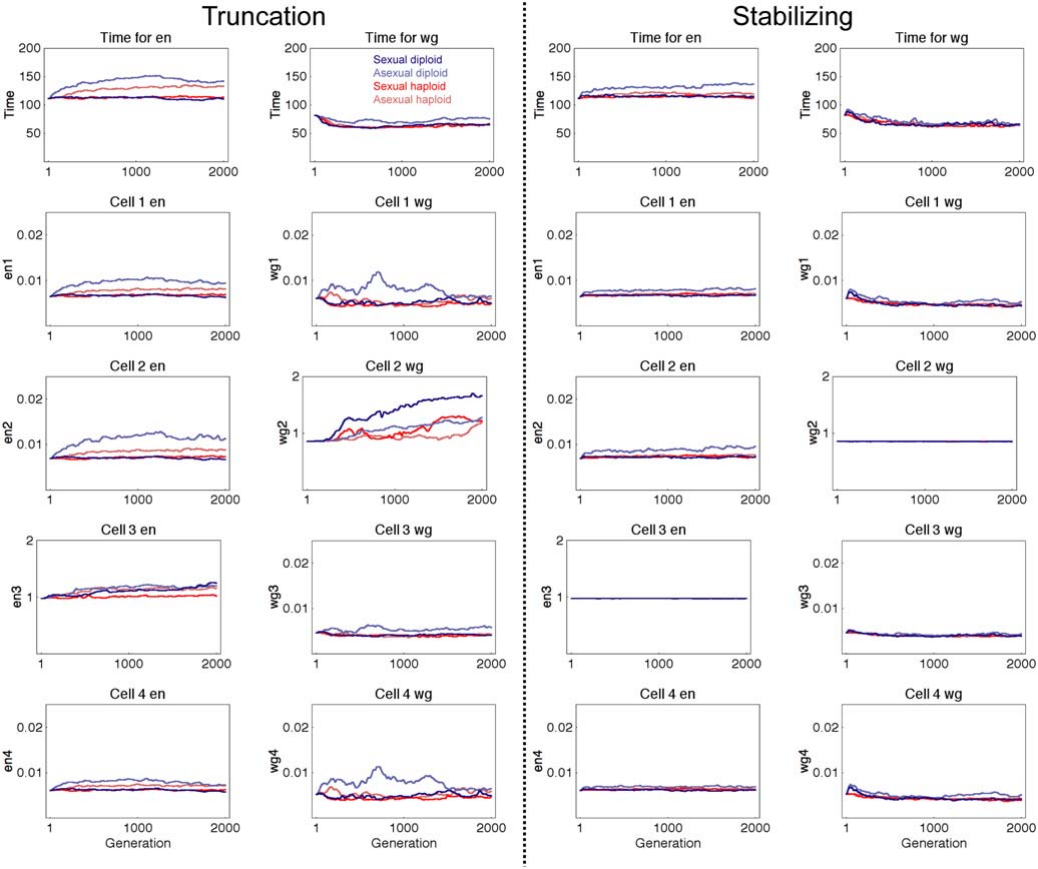
Evolution of phenotype. Comparison of changes in phenotype during evolutionary simulations. Plots show average of 40 trials, smoothed with a sliding window over 50 generations. Top row indicates the average time when all 4 cells satisfied the criteria in Figure 1 for wg (right column) and en (left column) expression. Remaining graphs show average en and wg expression level in each of the 4 cells from 200 to 500 simulated minutes of development. Cell 1 corresponds to the leftmost cell in the row of 4 cells from Figure 1C. The correct pattern is high en expression in cell 3 and high wg in cell 2 and low expression everywhere else. Expression levels were almost unchanged under stabilizing selection.

Under truncation selection, individuals were dead if they failed to sharpen en and wg sufficiently or if they did so too slowly. Populations under stabilizing selection were penalized if the en or wg pattern was altered, but fitness was independent of the time the prepattern was sharpened. To explore how aspects of the phenotype and network function evolved, Figure 7 plots the time at which the pattern was sharpened sufficiently and the average wg and en levels at the time selection acted (200–500 min). Under both selection types, populations evolved to sharpen wg more rapidly, with all populations showing similar speeding. In contrast, sexual populations maintained the time to sharpening of the en pattern, but asexual populations (particularly diploid asexual) showed slowed sharpening. Thus, evolution did not exclusively favor faster sharpening. Under truncation selection, the expression levels at which both wg and en stabilized (in the different cells that should express those genes highly) evolved to higher and higher values. Expression of wg showed more change compared to en, and wg expression often decreased in cells that had to express it at low levels. The highest possible en and wg level is 20 in our simulations, and requires both maximal transcription and highly stable products (long mean lifetimes). In general, diploid sexual populations show the greatest tendency to move away from thresholds of failure (high expression in the appropriate cell, and low elsewhere), while diploid asexual populations sometimes move towards expression thresholds (higher expression in cells that should express low levels, and slightly later en sharpening).

**Figure 8:**
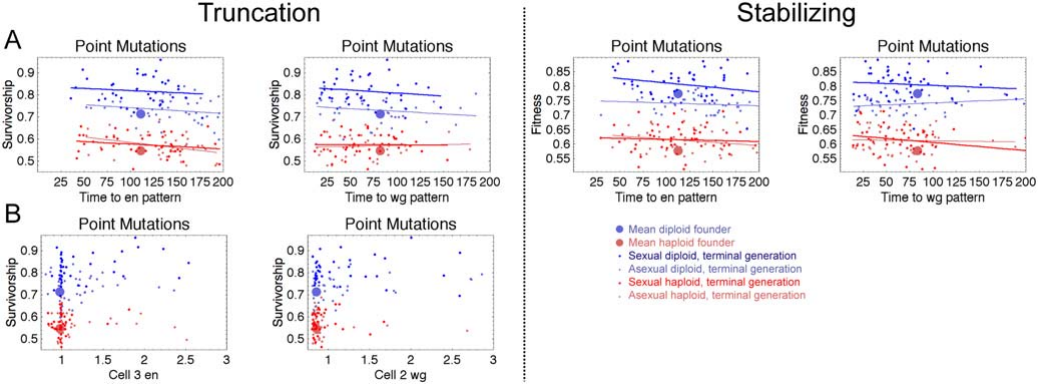
Correlation between phenotype and robustness. Plots show relationship between robustness and phenotype in each of the evolved populations. Large circles indicate the mean phenotype and robustness of the diploid and haploid founders. Small points show the phenotype and robustness at generation 2,000 for each of the 40 simulations. (A) Robustness to point mutations compared with the time to sharpening wg (right) and en (left) patterns. Lines are least-squares best fit to the generation 2,000 phenotypes, and show a weak increase in robustness with faster sharpening. (B) Robustness to point mutations compared with the wg (right) and en (left) level in the cell with high expression. Plot not shown for stabilizing selection, as there was little variation in en and wg expression (Figure 7). Lines were not fit to these data because the points for extreme values would dominate the fit. There may be a weak increase in robustness due to higher en and wg expression, but most populations did not dramatically change expression and it is clear the evolved robustness increase is not due solely to higher expression of en and wg.

Moving away from thresholds of failure could confer increased robustness by buffering the system to tolerate to small changes in expression. However, we emphasize the segment polarity network has been shown to exhibit highly nonlinear behavior, with successfully larger perturbations first producing almost no change in the pattern of expression followed by an abrupt collapse of the normal pattern [2]. Each of the 40 founders evolved slightly different phenotypes and robustness, and Figure 8 shows the correlation between the phenotype and robustness. Figure 8A plots mutational robustness against time to stabilization of the en and wg patterns for both truncation and stabilizing selection after 2,000 generations. Faster stabilization of the pattern was associated, on average, with only a modest increase in robustness. The average robustness of the diploid and haploid founders is also plotted (large circles). The best-fit lines indicate the correlation between evolved robustness and sharpening time; intersection of this line with the mean founder behavior indicates the robustness increase was due solely to changes in expression time. However, the best-fit lines lie above the founders, indicating that the robustness evolved through a mechanism independent of a faster time to sharpening. Similarly, Figure 8B correlates mutational robustness with expression level in the highest-expressing cell for truncation selection; there was little expression change under stabilizing selection. We did not fit lines to the data, as such a fit would be dominated by the outliers; most of the simulations showed little change in expression. However, there is only weak correlation between expression level and robustness, and the robustness that evolves is clearly not due solely to superthreshold buffering. Thus, both stabilization and truncation selection evolves greater robustness, particularly diploid sexual populations through mechanisms that do not have profound changes in phenotype.

### Diploid Sexual Populations Evolve Greater Robustness at Lower Mutation Rates

**Figure 9:**
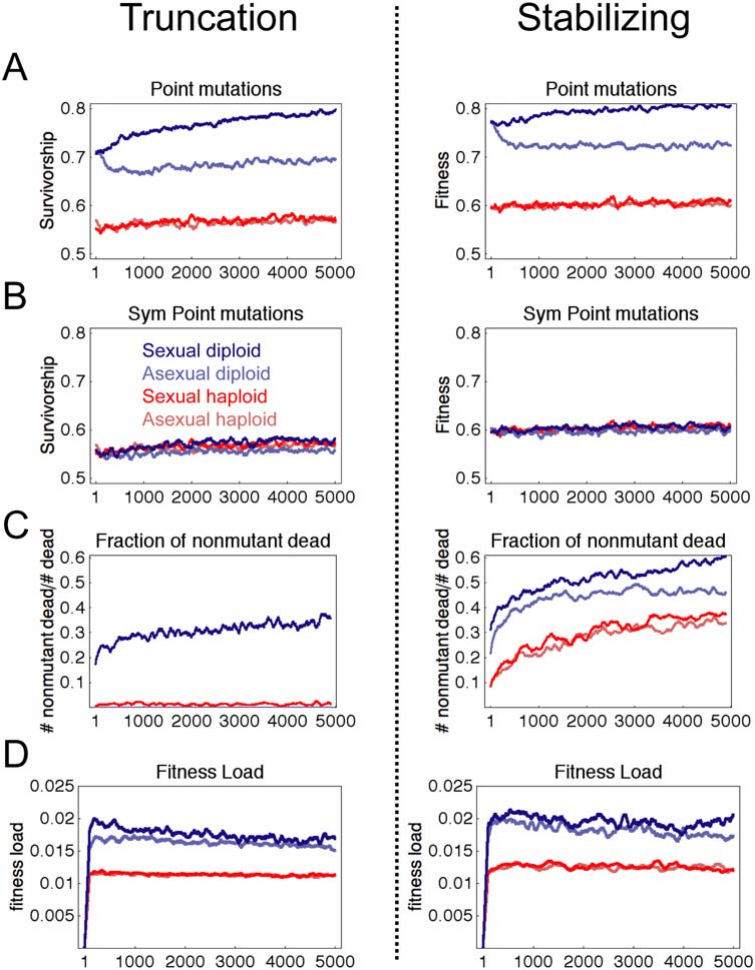
Diploid sexual populations evolve mutational robustness at lower mutation rate. Plots show results of 38 simulations to 5,000 generations with μ = 0.005, smoothed with a sliding window of 100 generations. (A) Robustness to point mutations shows diploid sexual populations evolve greater robustness, while diploid asexual populations have a transient decrease in robustness. (B) Symmetric double mutations were simulated the same as Figure 5D and eliminated most of the diploid robustness advantage. (C) Fraction of mutated individuals that were viable during evolutionary simulation. (D) Fitness load calculated according to Equation 16.

In the previous simulations, mutational robustness was expected to evolve due to the high mutation rate. Theory predicts such robustness should evolve when there is substantial genetic diversity, specifically when μN>1. Sex may allow selection for robustness at lower mutation rates, and this has been shown in randomly-wired transcriptional networks [35]. To test whether this holds in our network, we ran simulations with μ = 1/N = 0.005. Figure 9 shows the results of this simulation for 38 founders. We observed little robustness evolution in haploid populations, with no significant increase in robustness by generation 5,000. In contrast, diploid sexual populations evolved higher mutational robustness, while asexual diploid populations showed a transient decrease in robustness that stabilized by generation 1,000. As before, symmetric double mutations eliminated the diploid robustness advantage, indicating the diploid advantage was due to dominance of functional alleles. Again, recombination resulted in a greater fitness penalty in diploid populations compared to haploid (Figure 9C), and diploid sexual populations had the highest fitness load (Figure 9D). Thus, diploid sexual populations still experience the strongest selection for robustness when μN = 1.

## Discussion

We explored how ploidy and sex shape the evolution in a model of an actual, well-characterized, developmental genetic network. The segment polarity network is one of the best characterized networks, and comprises a functional module [2] that is conserved across insects and beyond. Previous theoretical and modeling studies have predicted mutational robustness can evolve, but we believe it is essential to test these findings in as detailed a model as possible. Our model allows us to bridge genotype to phenotype and to capture fundamentally important aspects of allelic fitness which no previous model has represented. We found that diploidy and sex combine to allow populations to evolve the greatest robustness to mutation, global perturbations affecting all interactions, and initial conditions. Diploidy confers an immediate robustness advantage as most deleterious mutations are recessive in our network, and over time the network evolves so that functional alleles become more dominant. Recombination, especially in diploid populations, produced a greater fitness load that selected for greater robustness evolution even at lower mutation rates of μ = 1/N. Recombination in our network constantly shuffled alleles and prevented the stabilization of matched allele combinations that could be maintained in asexual populations. In sexual populations, the constant shuffling of alleles by recombination in a genetically diverse population selects for those alleles that are highly compatible with others—i.e. alleles that are robust to genetic variation and mutation.

It is useful to compare our evolutionary model to that of previous computational studies on robustness evolution. Wagner [5] simulates evolution in randomly-wired haploid regulatory networks, which have been used in numerous studies [35],[36],[51],[52]. The Wagner model assumes a fixed time step, steep nonlinearities that result in effectively discrete expression levels and additive regulatory effects. All parameters reflect the strength of transcriptional activation/repression and mutation allows single mutations to change an inhibitor to an activator with 50% probability. In contrast to this, our model allows continuously variable expression levels with more graded nonlinearities (the maximum Hill coefficient in our simulations was 10). Previous models with fixed time steps [5],[36] reported a dramatic increase in speed in generating the target pattern of gene expression, while we observed only a slight speeding of wg (but not en) sharpening. In our model, molecular half lives can be mutationally altered as they would be in real life, which is difficult to translate to a fixed-time step model. It also permits non-additive interactions between multiple transcriptional regulators. Our model does not allow the sign of a regulatory interaction to change (inhibitors never switch to activators), and allows mutations to be *cis* or *trans* (the Wagner model parameters represent *cis* effects only [5]), thus allowing a meaningful exploration of diploidy. In our model, mutations are qualitatively similar to Wagner [5], as most result in nonproductive/weak interactions.

Other simulations have attempted to capture more accurate quantitative effects of mutation and other biological parameters. Robustness evolution has also been explored in models of mRNA secondary structure prediction [33],[34]. These models allow the detailed quantitative prediction of effects of mutation which we cannot do for our model; additionally it is difficult to explore the effects of recombination and diploidy in these models in a way that would meaningfully translate to genetic regulatory networks. It would be possible to alter our model so that the bit sequences represented mutable DNA/RNA sequences from which the interaction strength is calculated. We did not explore this because we wanted a general scheme to capture interactions within the network, and most model parameters reflect protein-protein or protein-DNA interactions. If we replaced our binary bit sequences with sequences of DNA bases (ATGC) or the 20 amino acids, there is no tractable function to describe how well the two would interact, as such a calculation would require prediction of the tertiary or quaternary structure. For the case of protein-DNA interactions with known binding motifs, the effects of mutations can be approximated [53],[54], and it would be an interesting extension to this work to incorporate a similar approximation. However, there are many interactions in addition to transcriptional regulation in our model, and such a scheme would not allow us to model all parameters. One final limitation of computer simulation is that we are limited by available computing power to relatively small populations and high mutation rates. Real *Drosophila* effective population sizes and mutation rates differ from our simulations by more than an order of magnitude. *Drosophila* populations are monomorphic for most genes (Nμ<1), so robustness is unlikely to evolve through the mechanism in our model. The small population size we use strongly increases the effect of drift, and may lead to increased genetic load and heterozygosity compared to larger/infinite populations [55]. Additionally, the increased drift due to low population sizes can hide the effect of weak selective pressures [56],[57]. Despite these limitations, our simulation incorporates a more realistic network and mutational effects than those in previous studies, and further advances in computing power will allow larger simulations.

Theory has predicted that sex and diploidy can evolve increased robustness in the presence of genetic variation [14], [27]–[29],[31],[32],[37]. Mutational robustness can evolve without recombination when there is sufficient genetic variation (Nμ>1). In randomly-wired haploid transcriptional networks, recombination leads to evolution of robustness when Nμ = 1 [35], a result that we did not observe in our haploid segment polarity network, though this may be due to the short duration of our simulations (5,000 generations) or small population sizes. More generally, Proulx and Phillips[14] predict that selection for robustness depends on the fitness load (effect of variation from all sources), and we clearly see diploid sexual populations have the greatest load (from mutation and recombination), while sex has little effect on haploid populations (Figures 6 and 9). Our results are generally consistent with this theory except for the substantial decrease in mutational robustness under conditions of lower mutation rate in asexual populations (Figure 9). The most likely explanation for this decrease is because the diploid founders are homozygous for all alleles, and thus both ‘halves’ of the network are identical. Theory predicts networks would rapidly accumulate deleterious recessive mutations[14],[58] that were masked by the working counterpart, and such mutations would persist in asexual populations without recombination to remove them. Because there is only a single working allele, there is nothing to rescue this network when that allele is mutated, resulting in a decrease in robustness compared to the founder. The decrease in robustness does not continue forever, reaching a minimum by approximately generation 1,000. The initial decrease in robustness reflects the loss of functionally redundant alleles possessed by the founders, consistent with theory that suggests selection to maintain both alleles is weak [58].

We found diploidy confers a robustness advantage primarily because most deleterious mutations are recessive to their working counterparts. Our model allows the possibility of dominant mutations, but predicts that most deleterious mutations are recessive in the segment polarity network. This is consistent with metabolic networks, however, we do not allow for the possibility of interference between two alleles (i.e. that wg1 might bind nonproductively to its targets, blocking wg2 activity as shown in Figure 3 and discussed in the Models section). Because of this, our model may underestimate the rate of dominant deleterious mutations, which are important for dominance evolution [59]. Future studies could explore the effect of more detailed allelic interaction, and incorporate more realistic rates of the different types of mutation and their quantitative effect, once such data is available. Additionally, our scheme allows us to simulate the effects of both *cis* and *trans* mutations, and future studies could also explore differences in mutational rates and whether they are consistent with genomic data [60].

The selection pressure that acts upon the real segment polarity network is not known. Since the segment polarity network stabilizes stripes of gene expression that activate downstream processes at the proper location, fitness must depend on the pattern produced. Our truncation selection explores the simple assumption that the expression of a segment polarity gene must be above a threshold for activation of those processes in the correct location and below this threshold everywhere else, for development to proceed normally. Alternatively, genomic data [45]–[47] suggest that many genetic networks are under stabilizing selection—maintaining specific, optimal levels of gene expression through time. Our simulations show truncation selection leads to evolution of higher gene expression (far above threshold) in those cells that should express the gene. Intuitively, very high gene expression levels should buffer the system to tolerate perturbations that cause slight changes in expression level [61], and our simulations are consistent with this intuition and previous modeling [62]. We do not impose a cost associated with higher expression, though presumably greater synthesis comes with a metabolic cost that would eventually limit the expression. The ultimate level of expression depends upon on the balance of synthesis and degradation, and mutations that solely increase the stability of a gene product likely have little metabolic cost, but it is difficult to determine the upper limit for gene product stability. Thus, in truncation selection, our founders had non-optimal patterns of gene expression that satisfied the developmental task; and evolved towards a more optimal phenotype with high expression levels of essential genes. However, the increase in expression alone shows only a weak correlation with increased robustness (Figure 8), and robustness in both truncation and stabilizing selection shows similar increases despite an unchanging pattern under stabilizing selection. Many parameters in our model reflect the activity of a gene product (*K* parameters) and so gene activity can change without changes in expression. It is likely that the absolute expression level is less important than the amount by which the expression exceeds the minimum/maximum threshold for activity. Thus, populations rapidly produce increased robustness regardless of whether the initial phenotype is optimal, and can evolve increased robustness without dramatic changes in phenotype.

Several extensions of this work warrant future study. We do not allow for the possibility of new regulatory interactions or gene duplication events (but we do allow for interaction loss) that alter the topology of the network. The topology of the segment polarity network to robustly stabilize stripes of wg and en expression may be nearly optimal, as indicated by a search of nearby network topologies in a simplified network [63]. It would be interesting to extend our simulations to allow the topology to change (i.e. the rise of new regulatory interactions) and gene duplication events, to see whether this topology is evolutionarily preserved or, if evolution settles on an alternate network. Gene duplication events would be particularly interesting because a duplication of all genes in the network would effectively increase the ploidy. Many organisms exist as tetraploid, octaploid or beyond, and others can amplify their genomic content through endoreplication [64] to attain very high ploidy(>1000C). Additionally, some viruses can have high effective ploidy when multiple viruses infect the same cell [65]. Our study suggests that having 2 copies of each gene can confer a robustness advantage over just one because most mutations are recessive and this more than compensates for the doubling of mutation rate. It would be interesting to explore under what conditions an increase in ploidy ceases to be advantageous in real networks, and why diploidy, as opposed to tetraploidy or beyond is so common. Finally, our scheme for translating genotype to model parameters would easily extend to randomly-wired networks used in previous studies [5], and allow diploid networks to be explored.

It is an open question as to how general our results are for other real networks. Theory and modeling studies indicated that increased phenotypic robustness readily evolves under conditions of interacting genes and variation in haploid networks [5],[31],[35],[36]. Based on these studies and ours, we speculate diploid sexual populations will evolve greater mutational robustness in networks when most deleterious mutations are recessive, and there is sufficient interaction between gene products so that recombination will select for alleles that can combine robustly with other alleles. By allowing both masking (by diploidy) and allele shuffling (recombination), the two can combine to achieve greater robustness than either alone. Thus, a sexual population for which robustness is important would likely favor a dominant diploid, not haploid, life cycle.

## Supporting Information

Protocol S1 List of equations in the model
(0.20 MB DOC)

Table S1 Relation between genotype and parameter values in model
(0.04 MB DOC)

Table S2 Detailed listing of all parameters in haploid model and range explored for random search for founders and during evolutionary simulation
(0.10 MB DOC)
