# Supplementary material for: Effects of Ploidy and Recombination on Evolution of Robustness in a Model of the Segment Polarity Network
Source: PLoS Comput Biol. 2009 Feb 27;5(2):e1000296. doi: 10.1371/journal.pcbi.1000296 (PMC2637435; doi:10.1371/journal.pcbi.1000296)
Supplement: Protocol S1 — List of equations in the model (0.20 MB DOC) [file pcbi.1000296.s001.doc]

**Protocol S1: List of equations in the model**

**List of haploid equations:**

Notation:

|  | (1) |
| --- | --- |
|  | (2) |
|  | (3) |
|  | (4) |
|  | (5) |
|  | (6) |
|  | (7) |
|  | (8) |
|  | (9) |
|  | (10) |
|  | (11) |
|  | (12) |
|  | (13) |

**List of diploid equations:**

|  | (14) |
| --- | --- |
|  | (15) |
|  | (16) |
|  | (17) |
|  | (18) |
|  | (19) |
|  | (20) |
|  | (21) |
|  | (22) |
|  | (23) |
|  | (24) |
|  | (25) |
|  | (26) |
|  | (27) |
|  | (28) |
|  | (29) |
|  | (30) |
|  | (31) |
|  | (32) |
|  | (33) |
|  | (34) |
|  | (35) |
|  | (36) |
|  | (37) |
|  | (38) |
|  | (39) |
|  | (40) |
|  | (41) |
