# Supplementary material for: Effects of Ploidy and Recombination on Evolution of Robustness in a Model of the Segment Polarity Network
Source: PLoS Comput Biol. 2009 Feb 27;5(2):e1000296. doi: 10.1371/journal.pcbi.1000296 (PMC2637435; doi:10.1371/journal.pcbi.1000296)
Supplement: Table S1 — Relation between genotype and parameter values in model (0.04 MB DOC) [file pcbi.1000296.s002.doc]

**Table S1**: Relation between genotype and parameter values in model

| Parameter Prefix | Meaning | Calculated from: |
| --- | --- | --- |
| *C* | Max transcription rate | gene property compared to 0 |
| *L* | Max translation rate | gene property compared to 0 |
| *H* | Mean lifetime | gene property compared to 0 |
| *K* | Half maximal activation coefficient | complimentarity between 2 interacting genes |
| *ν* | Hill coefficient | gene property compared to 0 |
| *κ* | Dimerization rate | complimentarity between 2 interacting genes |
| *α* | saturability coefficient for an enhancer | gene property compared to 0 |
| *r* | transfer/transform rates | gene property compared to 0 |
