# Supplementary material for: Effects of Ploidy and Recombination on Evolution of Robustness in a Model of the Segment Polarity Network
Source: PLoS Comput Biol. 2009 Feb 27;5(2):e1000296. doi: 10.1371/journal.pcbi.1000296 (PMC2637435; doi:10.1371/journal.pcbi.1000296)
Supplement: Table S2 — Detailed listing of all parameters in haploid model and range explored for random search for founders and during evolutionary simulation (0.10 MB DOC) [file pcbi.1000296.s003.doc]

**Table S2**: Detailed listing of all parameters in haploid model and range explored for random search for founders and during evolutionary simulation. Second column (genes involved in interaction) lists whether the parameter reflects an interaction between the gene product and cellular machinery (if only 1 gene listed) or an interaction between two segment polarity genes (2 genes listed). For parameters with 1 gene listed, there are 2 versions of the parameter in the diploid model; for parameters with 2 genes listed, there are 4 versions of the parameter in the diploid model. ^*^ This parameter was calculated using Equation 8

| Parameter | Genes involved in interaction | Random search range (min-max) | Evolutionary range (min-max) | Sampling |
| --- | --- | --- | --- | --- |
| *C_en_* | en | 1/*H_en_*-1/*H_en_* | 1x10^-8^ - 0.2 | Log |
| *L_EN_* | en | 1/*H_EN_*-1/*H_EN_* | 1x10^-8^ - 0.2 | Log |
| *H_en_* | en | 5-100 | 1x10^-6^ - 100 | Log |
| *H_EN_* | en | 5-100 | 1x10^-6^ - 100 | Log |
| *C_wg_* | wg | 1/*H_wg_*-1/*H_wg_* | 1x10^-8^ - 0.2 | Log |
| *L_WG_* | wg | 1/*H_EWG_*-1/*H_EWG_* | 1x10^-8^ - 0.2 | Log |
| *H_wg_* | wg | 5-100 | 1x10^-6^ - 100 | Log |
| *H_IWG_* | wg | 5-100 | 1x10^-6^ - 100 | Log |
| *H_EWG_* | wg | 5-100 | 1x10^-6^ - 100 | Log |
| *C_ptc_* | ptc | 1/*H_ptc_*-1/*H_ptc_* | 1x10^-8^ - 0.2 | Log |
| *L_PTC_* | ptc | 1/*H_PTC_*-1/*H_PTC_* | 1x10^-8^ - 0.2 | Log |
| *H_ptc_* | ptc | 5-100 | 1x10^-6^ - 100 | Log |
| *H_PTC_* | ptc | 5-100 | 1x10^-6^ - 100 | Log |
| *C_cid_* | cid | 1/*H_cid_*-1/*H_cid_* | 1x10^-8^ - 0.2 | Log |
| *L_CID_* | cid | 1/*H_CID_*-1/*H_CID_* | 1x10^-8^ - 0.2 | Log |
| *H_cid_* | cid | 5-100 | 1x10^-6^ - 100 | Log |
| *H_CID_* | cid | 5-100 | 1x10^-6^ - 100 | Log |
| *H_CN_* | cid | 5-100 | 1x10^-6^ - 100 | Log |
| *C_hh_* | hh | 1/*H_hh_*-1/*H_hh_* | 1x10^-8^ - 0.2 | Log |
| *L_HH_* | hh | 0.2/*H_HH_*-5/*H_HH_* | 1x10^-8^ - 1 | Log |
| *H_hh_* | hh | 5-100 | 0.5-100 | Log |
| *H_HH_* | hh | 5-100 | 1x10^-6^ - 100 | Log |
| *H_PH_* | hh, ptc | 5-100 | 1x10^-6^ - 100 | Log ^*^ |
| *r_EndoWG_* | wg | 0.001-1 | 1x10^-7^ -1 | Log |
| *r_ExoWG_* | wg | 0.01-10 | 1x10^-7^ -10 | Log |
| *r_MxferWG_* | wg | 0.001-1 | 1x10^-7^ -1 | Log |
| *r_LMxferWG_* | wg | 0.001-1 | 1x10^-7^ -1 | Log |
| *r_LMXferPTC_* | ptc | 0.001-1 | 1x10^-7^ -1 | Log |
| *r_LMxferHH_* | hh | 0.001-1 | 1x10^-7^ -1 | Log |
| *κ_PTChh_* | ptc, hh | 0.001-1 | 0.001-100 | Linear |
| *K_PTCcid_* | ptc, cid | 0.001-1 | 0.001-100 | Linear |
| *ν_PTCcid_* | cid | 1-10 | 1-10 | Log Integer |
| *m_PTCcid_* | cid | 0.001-1 | 1x10^-7^ -1 | Log |
| *K_EWGen_* | wg, en | 0.001-1 | 0.001-100 | Linear |
| *ν_EWGen_* | en | 1-10 | 1-10 | Log Integer |
| *K_CNewg_* | cid, wg | 0.001-1 | 0.001-100 | Linear |
| *ν_CNewg_* | wg | 1-10 | 1-10 | Log Integer |
| *K_IWGwg_* | wg, wg | 0.001-1 | 0.001-100 | Linear |
| *ν_IWGwg_* | wg | 1-10 | 1-10 | Log Integer |
| *α_IWGwg_* | wg | 1-10 | 1-10 | Linear |
| *K_CIDwg_* | cid, wg | 0.001-1 | 0.001-100 | Linear |
| *ν_CIDwg_* | wg | 1-10 | 1-10 | Log Integer |
| *α_CIDwg_* | wg | 1-10 | 0.1-10 | Log |
| *K_WCNcid_* | cid, cid | 0.001-1 | 0.001-100 | Linear |
| *ν_WCNcid_* | cid, cid | 1-10 | 1-10 | Log Integer |
| *K_CIDptc_* | cid, ptc | 0.001-1 | 0.001-100 | Linear |
| *ν_CIDptc_* | ptc | 1-10 | 1-10 | Log Integer |
| *K_CNcid_* | cid, cid | 0.001-1 | 0.001-100 | Linear |
| *ν_CNcid_* | cid | 1-10 | 1-10 | Log Integer |
| *K_Bcid_* | cid | 0.001-1 | 0.001-100 | Linear |
| *ν_Bcid_* | cid | 1-10 | 1-10 | Log Integer |
| *K_ENb_* | en | 0.001-1 | 0.001-100 | Linear |
| *ν_ENb_* | en | 1-10 | 1-10 | Log Integer |
| *K_ENhh_* | en, hh | 0.001-1 | 0.001-100 | Linear |
| *ν_ENhh_* | hh | 1-10 | 1-10 | Log Integer |
| *K_CNen_* | cid, en | 0.001-1 | 0.001-100 | Linear |
| *ν_CNen_* | en | 1-10 | 1-10 | Log Integer |
